# Through the Gap: A Rare Case of Intercostal Lung Hernia

**DOI:** 10.7759/cureus.81230

**Published:** 2025-03-26

**Authors:** Samarth Arora, Aditi H Dongre, Priscilla Joshi

**Affiliations:** 1 Radiology, Bharati Vidyapeeth (Deemed to be University) Medical College and Hospital, Pune, IND

**Keywords:** intercostal lung hernia, lung contusion, lung hernia, lung laceration, open pneumothorax, rib fractures, stab injury, subcutaneous air, trauma, traumatic lung hernia

## Abstract

Intercostal lung hernia is a rare condition characterized by the protrusion of lung tissue through a defect in the intercostal muscles or thoracic wall. It can be congenital or arise secondary to trauma or surgery or can be a spontaneous rupture due to elevated intrathoracic pressure. Given its rarity, intercostal lung hernia can present a diagnostic and therapeutic challenge.

We present the case of a 36-year-old man who came to our hospital's Emergency Medicine Department with an alleged history of assault with a sharp object. He sustained a stab wound over the lateral aspect of the left side of the chest associated with bleeding from the puncture site. On assessment, there was decreased air entry on the left side of the chest. A supine chest radiograph, on admission, revealed a suspicious ill-defined lucency in the subcutaneous tissues of the left chest wall. He subsequently underwent a contrast CT of the thorax on a 128-slice CT machine which revealed lung tissue in the subcutaneous plane of the left chest wall suggestive of a lung herniation. The patient underwent surgery and is recovering well.

This case report highlights a unique presentation of intercostal lung hernia, emphasizing the need for early diagnosis and expeditious treatment.

## Introduction

Lung hernia is an uncommon clinical entity characterized by the protrusion of lung tissue beyond the thoracic cage [1]. Its rarity often poses diagnostic and therapeutic challenges primarily because it is often not considered in differential diagnoses. Its clinical features, such as chest pain, dyspnea, or a palpable bulge, are nonspecific and can mimic more common conditions like pulmonary embolism, pneumonia, or malignancies [2].

Lung herniation, though rare, was first described in 1499 by Roland [3] in a case which was supra-clavicular in location. Since then, more than 300 cases have been reported in the literature [4,5]. 

In 1845, Morel-Lavallée first classified pulmonary hernias based on their anatomical location into cervical, intercostal, or diaphragmatic [6]. Lung hernias were also classified according to their etiology into congenital, traumatic, and spontaneous [6].

Acquired lung hernias were most commonly caused by trauma, accounting for 52% of cases. Traumatic hernias were seen secondary to blunt or penetrating chest injuries, such as stab wounds or accidents involving heavy objects. However, they can also develop spontaneously in 30% of cases or as a result of underlying lung pathology in 18% of instances [7].

Spontaneous hernias were seen to occur due to increased intrathoracic pressure or pathological conditions that weakened the thoracic structures [6,8] with activities like weightlifting or severe coughing [1].

Chronic obstructive pulmonary disease (COPD) is a common comorbidity in patients with spontaneous lung herniation, likely due to chronic coughing and lung hyperinflation, which place excessive strain on the thoracic structures. Additionally, long-term steroid use in COPD patients may contribute to muscle and connective tissue weakening, further increasing the risk of herniation [9].

Other potential risk factors include male gender, a history of smoking, and obesity, all of which have been associated with reduced chest wall integrity and increased intrathoracic pressure, making individuals more susceptible to spontaneous lung herniation [10].

Although no specific weight threshold for weightlifting has been established, any activity causing significant intrathoracic pressure spikes can trigger a hernia, particularly in individuals with predisposing factors. Additionally, occupations involving heavy physical labor or chronic coughing may increase the risk. Recognizing these risk factors is essential for the early diagnosis and appropriate management of spontaneous lung hernias [11].

Symptoms present as chest pain that worsens with deep breathing or movement, difficulty breathing (dyspnea), and a persistent cough, which may sometimes be productive. In severe cases, strangulation of the lung tissue can occur, leading to intense pain, respiratory distress, and cyanosis. The most noticeable signs include a visible or palpable bulge that may change in size with breathing or coughing, crepitus (a crackling sensation under the skin due to trapped air), and reduced breath sounds over the affected area.

With the advent of cross-sectional imaging like CT scan, the ability to diagnose and further evaluate lung hernias with the visualization of the defect, surrounding structures, and lung tissue is possible, which was not seen on routine chest radiographs.

Lung herniation can manifest in various ways. Physicians should be aware of symptomatic cases that manifest as chest pain, dyspnea, or a palpable bulge, as these may lead to complications like incarceration or strangulation. However, many cases remain asymptomatic [12]. A research done in 2023 which analyzed 38 patients found that 33 were asymptomatic, suggesting that the majority do not require immediate intervention [12]. 

Asymptomatic cases may be managed conservatively, but symptomatic hernias or those at risk of complications, such as strangulation or respiratory distress, often necessitate surgical intervention [13].

## Case presentation

A 36-year-old man presented to the Emergency Medicine Department with an alleged history of stab injury to the left side of the chest following an assault with a sharp object on 19/11/2024 around 21:30 hours. He also had a lacerated wound in the right parieto-temporal region. There was no history of loss of consciousness, nausea, vomiting, nasal or oral bleeding, or seizures.

Following the assault, he was brought to our hospital for treatment by his uncle around three hours after the incident. 

The patient was a construction worker by occupation and belonged to a middle socioeconomic status. He was a recently diagnosed case of diabetes mellitus and hypertension, on Tab. Metformin and Tab. Met XL. He had a circumcision done two years back for personal reason. He had no known allergies or history of substance abuse. He was right-handed.

The patient was hemodynamically stable and afebrile with a pulse rate of 90/minute, a blood pressure of 130/90 mmHg, a respiratory rate of 16/minute, and an oxygen saturation (SpO2) of 97% on room air. Pallor, icterus, edema, and lymphadenopathy were absent. There was no neurological deficit, and the patient had a Glasgow Coma Scale (GCS) score of 15/15. No significant blood loss was noted at the time of admission and as per information provided by the patient and his attendant (Advanced Trauma Life Support (ATLS) Class I).

Respiratory examination revealed that the patient had decreased air entry on the left side and normal on the right.

**Figure 1:**
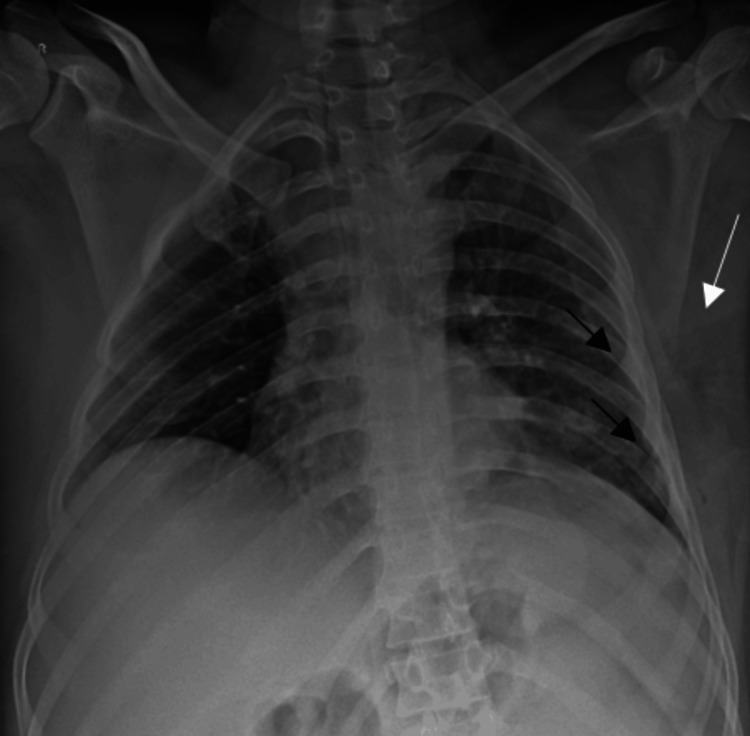
Chest radiograph taken in the supine position shows a few inhomogeneous opacities in the left middle and lower zones with a suspicious ill-defined lucency in the subcutaneous tissues of the left chest wall (white arrow). Linear displaced fractures of the left fourth and fifth ribs are also seen (black arrow).

In view of this, a chest radiograph obtained in the supine position revealed a few inhomogeneous opacities in the left middle and lower zones with a suspicious ill-defined lucency in the subcutaneous tissues of the left chest wall. Linear displaced fractures of the left fourth and fifth ribs were also seen (Figure 1).

Focused assessment revealed a clean-cut, deep stab wound of size 6 × 3 cm over the lateral aspect of the left chest (in front of the left axilla) associated with bleeding from the site of the wound. The significant depth of the wound raised suspicion of a possible open pneumothorax. Hence, a 28-French intercostal drain was placed in the left fifth intercostal space in the anterior axillary line, over the postero-lateral aspect of the left lung, and connected to a bag. Air-column movement was noted. The drain was successfully placed in the first attempt.

**Figure 2:**
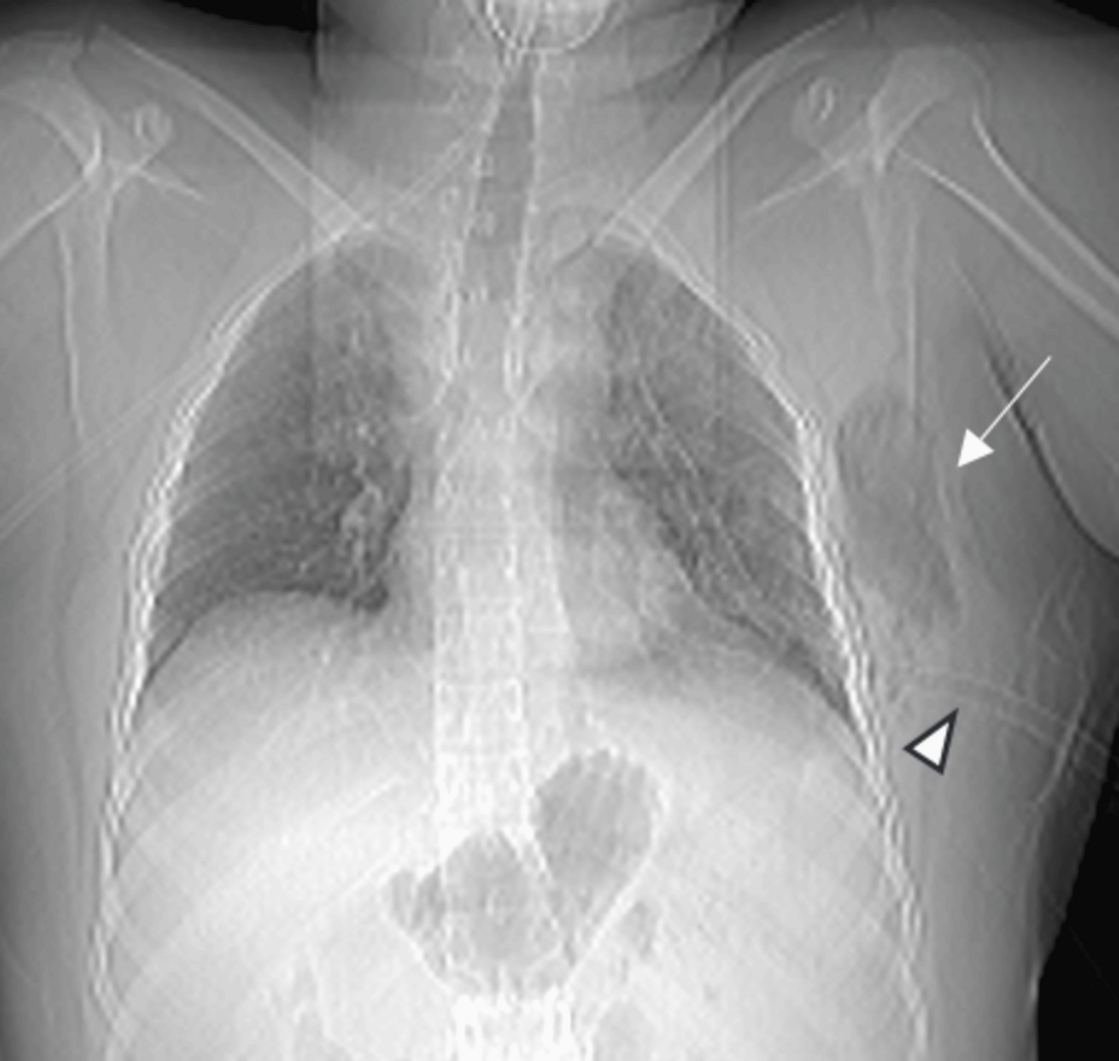
Scanogram image shows lucency in the subcutaneous tissues of the left chest wall (white arrow). ICD is seen on the left in the fifth intercostal space (arrowhead). ICD: implantable cardioverter defibrillator

**Figure 3:**
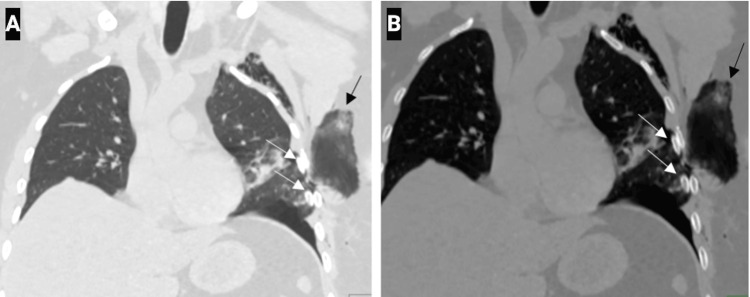
Coronal chest CT image with lung window (A) and bone window (B) setting shows lung herniation through a defect (black arrow) between the linear displaced fractures of the left fourth and fifth ribs (white arrows). Subcutaneous emphysema is also seen.

**Figure 4:**
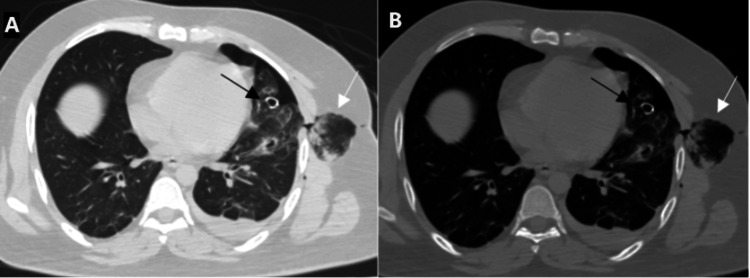
Axial chest CT image with lung window (A) and bone window (B) setting shows the loculated area of air density with lung markings within it in the subcutaneous plane of the left hemithorax (white arrow). This appears to be in continuity with the lung. Associated hemopneumothorax is also seen. The ICD is seen "end on" (black arrow). ICD: implantable cardioverter defibrillator

**Figure 5:**
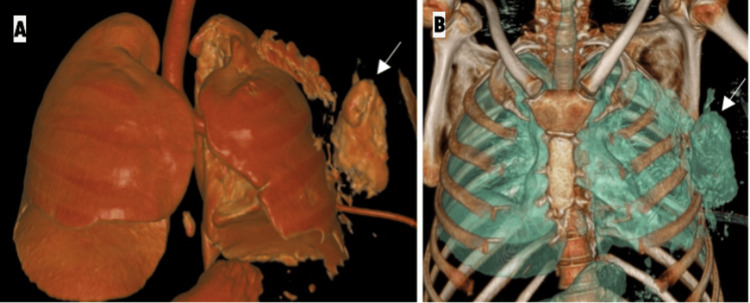
Volume-rendered three-dimensional reconstructed CT images (A and B) show the intercostal lung hernia through the left fourth intercostal space (white arrow).

A CT of the thorax with contrast was done to confirm the findings of the chest radiograph and revealed a loculated area of air density in the subcutaneous plane of the left hemithorax, appearing to be in continuity with the lung. It was observed that the air density had increased compared to the chest radiograph. This was suggestive of lung herniation through a defect between linear displaced fractures of the fourth and fifth ribs (Figures 2-5). Another possibility of a loculated subcutaneous emphysema was considered as a differential diagnosis.

Associated left hemopneumothorax with left lung volume loss, subcutaneous emphysema along the left chest wall, and contusions of the inferior lingular, superior, and lateral basal segments of the left lower lobe were also seen.

**Figure 6:**
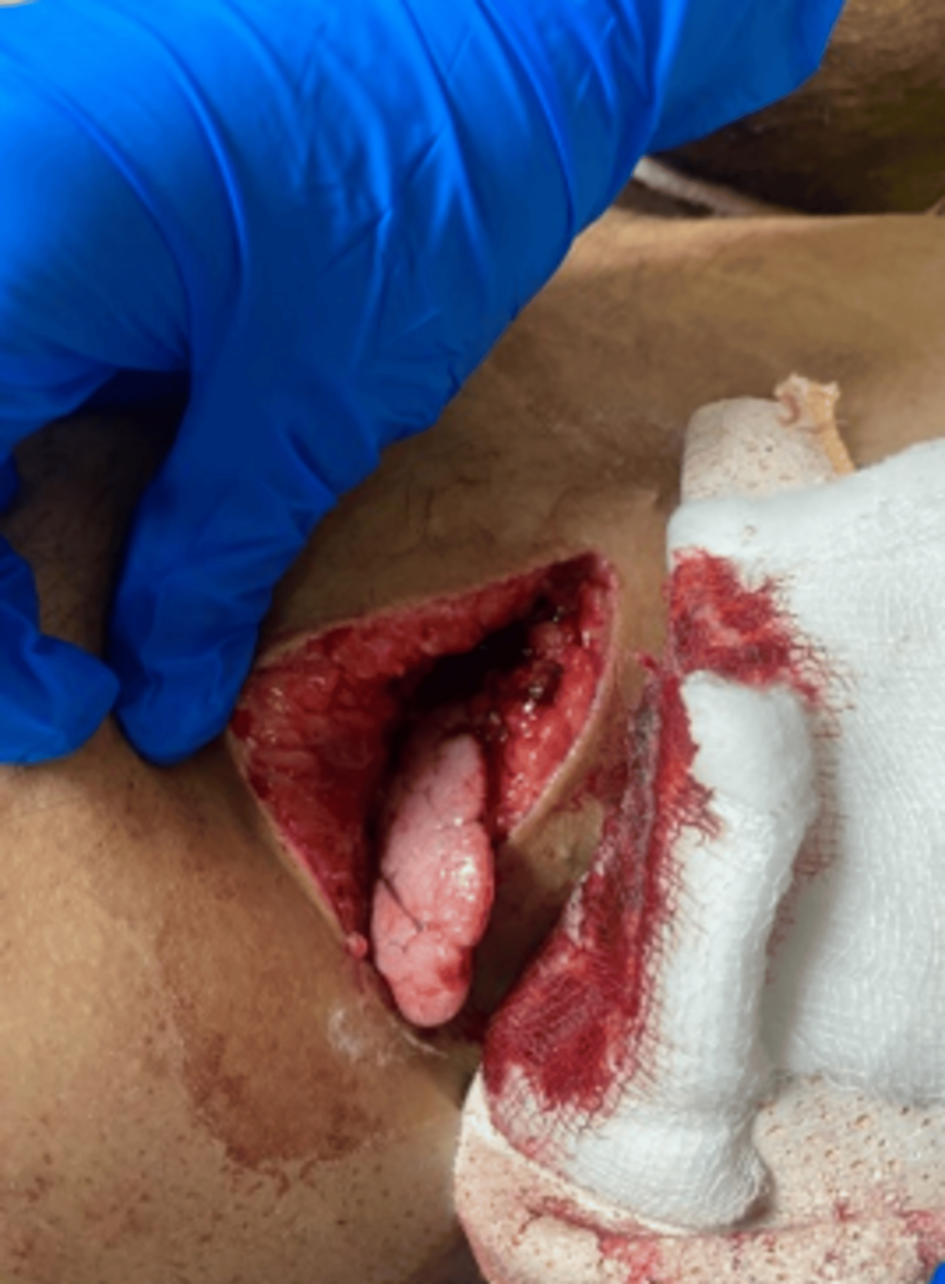
Pre-operative image shows the lung tissue herniating through the stab wound.

**Figure 7:**
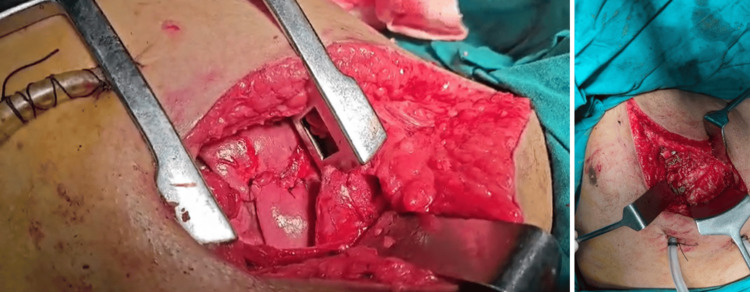
Intra-operative images show the repair of the left lung laceration with wiring of the left fourth and fifth rib fractures.

The patient was taken for emergency wound exploration, and repair of the left lung laceration with wiring of the left fourth and fifth rib fractures was done (Figure 6 and Figure 7). Hemostasis was ensured.

**Figure 8:**
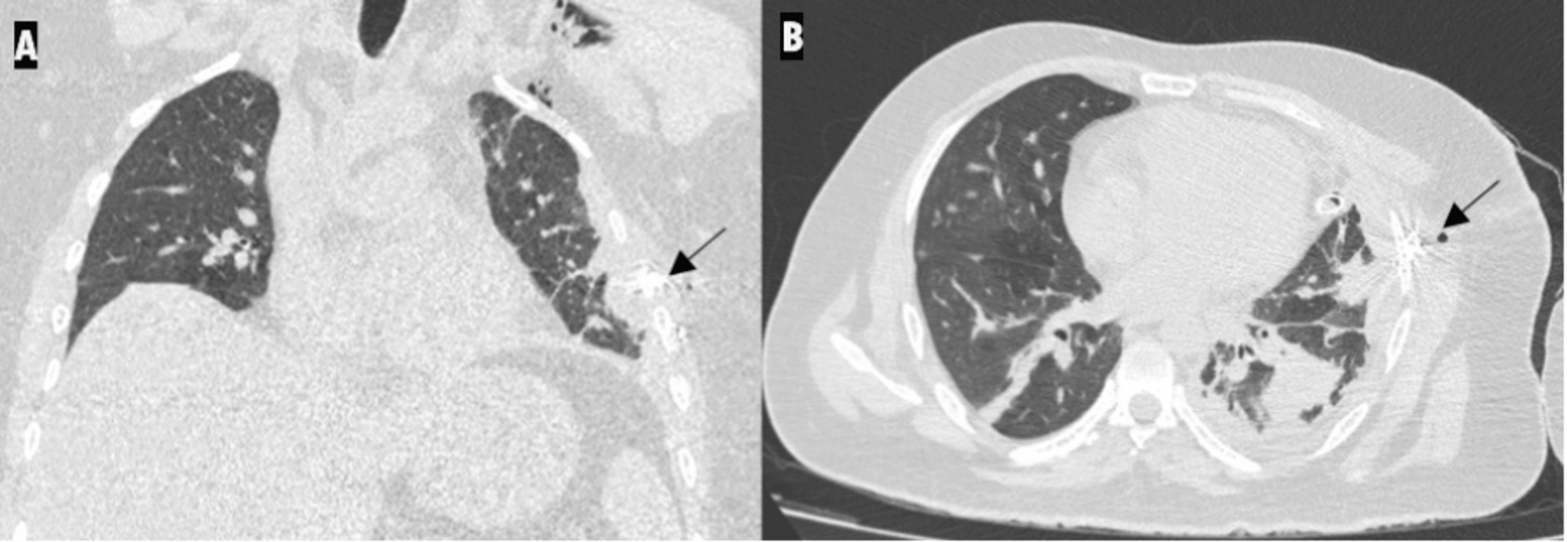
Coronal (A) and axial (B) chest CT images (post-operative day 4) show pleural collection with resolving contusions in the lingula and left lower lobe. Post-operative changes are seen in the left fourth and fifth ribs (black arrow). Residual subcutaneous emphysema is also noted. The ICD is seen "end on". ICD: implantable cardioverter defibrillator

**Figure 9:**
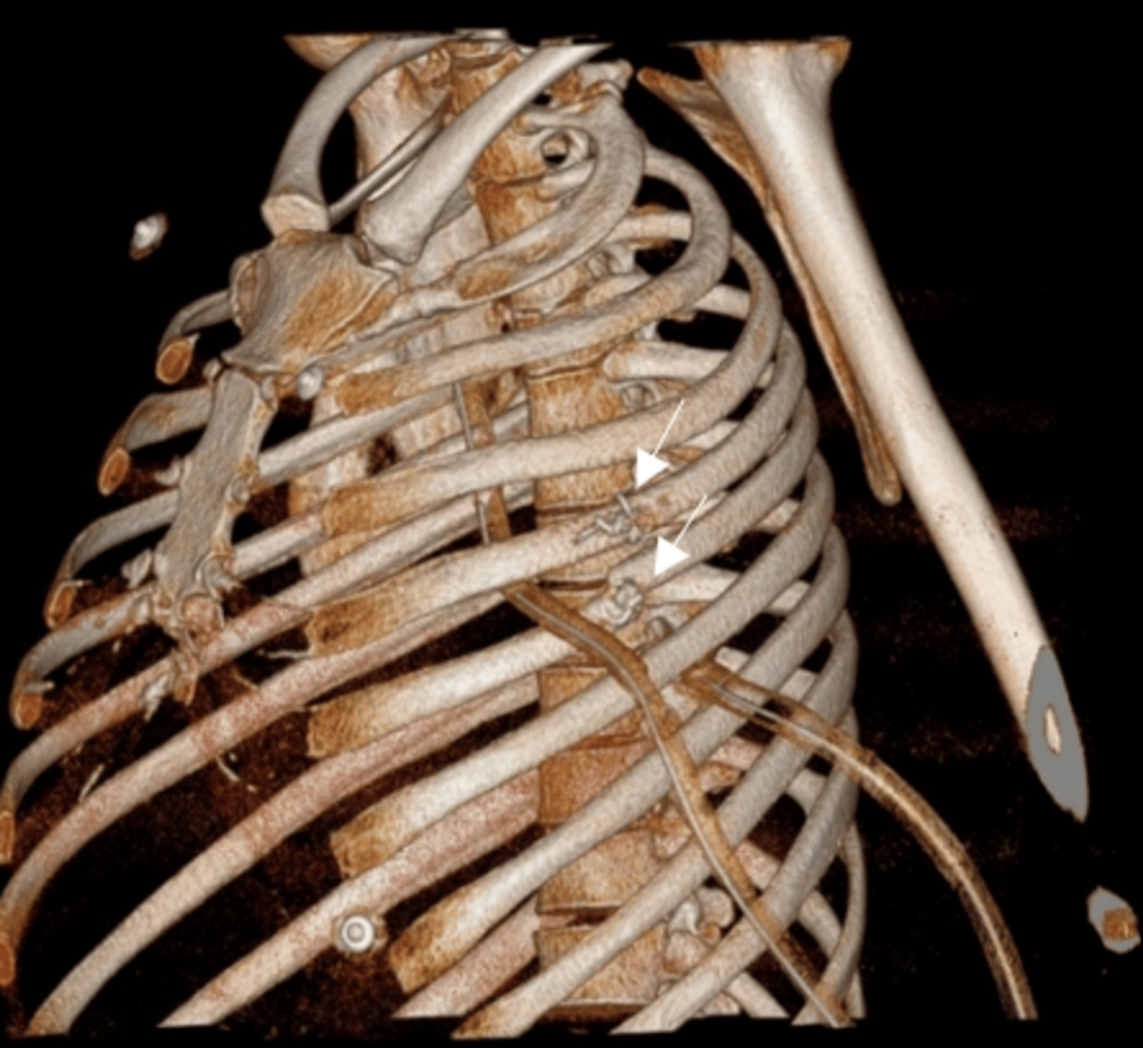
Volume-rendered three-dimensional reconstructed chest CT image shows the wiring of the fourth and fifth ribs (white arrows). ICD tubes are also seen. ICD: implantable cardioverter defibrillator

A repeat CT scan done after four days revealed mild residual pneumothorax and pleural collection with resolving contusions in the lingula and left lower lobe (Figure 8). Post-operative changes in the left fourth and fifth ribs were also seen (Figure 9).

The patient is in recovery and is doing well. He was shifted to the ICU for observation post-surgery for two days and shifted to the ward currently. He is afebrile, and bilateral air entry is present with a respiratory rate of 14/minute and SpO2 of 98% on room air. ICD output is nil.

## Discussion

This case illustrates a rare presentation of an intercostal lung hernia following a penetrating chest trauma, due to an injury sustained during an assault with a sharp object. A high index of suspicion is imperative especially in patients presenting with penetrating chest trauma and persistent respiratory symptoms since routine chest radiographs may often be inconclusive. Cross-sectional imaging with a CT scan is crucial for accurate diagnosis, as it provides detailed anatomical insights into the hernia's location and size and its relationship with surrounding structures.

In our case, the penetrating injury led to a defect in the intercostal musculature, allowing the lung tissue to herniate through the thoracic wall. Increased intrathoracic pressure during respiratory excursions and compromised thoracic wall integrity were key contributing factors for the gradual increase in the size of the hernia as evidenced by the change in the size of the lucency within the soft tissues overlying the left hemithorax evidenced on the radiograph and subsequent chest scanogram.

In the past, thoracic strapping was used to manage lung hernias, but this approach has been abandoned due to reduced pulmonary compliance, atelectasis, and infection. Early surgical repair now offers the best outcome, with low morbidity and excellent long-term prognosis [14].

Techniques vary, including repairs with or without prosthetic patches, and video thoracoscopic methods have been suggested [15].

However, no single approach suits all hernias, and management is individualized. Rib fractures, often associated with traumatic lung herniation, increase the risk of complications like lung tissue incarceration and would require repair or resection [16].

While small, uncomplicated hernias can be managed conservatively with fair outcomes, rib fractures in elderly patients with high peri-operative risk present a therapeutic challenge, sometimes necessitating non-surgical management [17].

## Conclusions

Intercostal lung hernia, though rare, should be considered in patients with chest trauma, especially those with a history of penetrating chest trauma who may be asymptomatic or presenting with respiratory complaints. CT imaging is indispensable for definitive diagnosis, guiding surgical planning, and identifying potential complications. Early surgical management offers favorable outcomes and minimizes the risk of severe complications.
